# Humans, fish, spiders and bees inherited working memory and attention from their last common ancestor

**DOI:** 10.3389/fpsyg.2022.937712

**Published:** 2023-02-06

**Authors:** Brian Earl

**Affiliations:** Independent Researcher, Melbourne, VIC, Australia

**Keywords:** working memory, attention, phylogenetic conservation, LCA of vertebrates and arthropods, brain evolution, evolution of mind, arthropod mind, vertebrate mind

## Abstract

All brain processes that generate behaviour, apart from reflexes, operate with information that is in an “activated” state. This activated information, which is known as working memory (WM), is generated by the effect of attentional processes on incoming information or information previously stored in short-term or long-term memory (STM or LTM). Information in WM tends to remain the focus of attention; and WM, attention and STM together enable information to be available to mental processes and the behaviours that follow on from them. WM and attention underpin all flexible mental processes, such as solving problems, making choices, preparing for opportunities or threats that could be nearby, or simply finding the way home. Neither WM nor attention are necessarily conscious, and both may have evolved long before consciousness. WM and attention, with similar properties, are possessed by humans, archerfish, and other vertebrates; jumping spiders, honey bees, and other arthropods; and members of other clades, whose last common ancestor (LCA) is believed to have lived more than 600 million years ago. It has been reported that very similar genes control the development of vertebrate and arthropod brains, and were likely inherited from their LCA. Genes that control brain development are conserved because brains generate adaptive behaviour. However, the neural processes that generate behaviour operate with the activated information in WM, so WM and attention must have existed prior to the evolution of brains. It is proposed that WM and attention are widespread amongst animal species because they are phylogenetically conserved mechanisms that are essential to all mental processing, and were inherited from the LCA of vertebrates, arthropods, and some other animal clades.

## 1. Introduction

The term “working memory” (WM) was proposed by Miller et al. (1960) in reference to the retention of plans for action, which they also referred to as “intentions”. They wrote (p. 59) that the plan is “put into some special state or place where it can be remembered… a kind of quick-access, ‘working memory’.” The concept of WM was extended by Atkinson and Shiffrin (1968) who included “active” information in short-term memory (STM) and long term memory (LTM).

The WM models of Miller et al. (1960) and Atkinson and Shiffrin (1968) were followed by the “multicomponent model” (Baddeley and Hitch, 1974). The multicomponent model had three parts: a verbal working memory (“phonological loop”), a visual–spatial working memory (“visuospatial sketchpad”), and the “central executive”, which included a system for controlling the focus of attention. This model was later modified to include a fourth component, the “episodic buffer”, which combined information from different sources in a form that is experienced (Baddeley, 2000, 2010).

According to this view, WM can include plans, incoming sensory information, and information from STM or LTM (Cowan, 1988, 1999). Cowan wrote that WM is “activated information”, which is the focus of attention, is associated with consciousness, and is used for quite complex operations, such as problem solving, decision-making, and other thought processes.

The Baddeley and Hitch multicomponent model was the dominant view of WM until recently. A move away from this view has occurred as a result of the many investigations of aspects of WM and attention, principally in humans, that has created a more detailed picture of their properties and the relationship between WM and attention. This has led to reports that WM and attention have properties which are very important for the survival of mobile animals. WM and attention help their possessors to determine the identities and movements of objects in their environment, and therefore to respond better to threats and opportunities. Following on from this, it has been suggested that WM and attention may have been early adaptations in the evolution of cognitive systems (Haladjian and Montemayor, 2015).

The current view is that WM is information which is retained by an organism—generally for a short time after an event—in an active state that makes it available for use in the generation of responses. WM enables a wide range of mental activities, from complex calculations to simply preparing to respond to significant objects—predators, prey, potential mates, and so on—that, for some reason are no longer sensed, but may still be nearby (Fuster, 1990; Wallis et al., 2015). WM is essential to us for all thinking, planning and problem-solving, and for simpler tasks such as not looking in the same place twice for a lost object.

Evidence is accumulating in support of the view that WM corresponds to neural activity in relevant areas of STM (Haynes and Rees, 2005; Kamitani and Tong, 2005; Offen et al., 2009; Serences et al., 2009; Chun, 2011; Lewis-Peacock et al., 2012) or LTM (Ruchkin et al., 2003; Lewis-Peacock and Postle, 2008). In each case, attention-related processes activate neurons that represent information concerning whatever is being attended to.

*Attention* (sometimes referred to as “selective attention”) is the occurrence in animals of enhanced “active” information relevant to a matter of concern (“the focus”), and reduced information concerning matters outside of the focus. Note that the focus is not necessarily a visual focus. This active information is WM, and is available for investigating a focus, and preparing a response to it (Olivers and Roelfsema, 2020). The focus may be important biologically (in any species), or socially or personally (in some species).

There is a reciprocal relationship between WM and attention. Information from the attentional focus becomes the content of WM, but attention tends to be drawn to objects that are related to the information held in WM. This is because WM is information that is available to mental processes, which would include processes that select the attentional focus.

Attentional processes select a focus from WM and other available information. The other available information is *preattention* (or “global perception”), from which meaningful items or locations can be selected for attention (Logan, 1992; Wolfe, 1992). It appears that preattention and its associated processes are ongoing independently of attention, so that significant items or changes are immediately noticed, they “pop out”, even when attention is directed elsewhere (Hughes et al., 2012). Any matter that was recently a focus of attention—about which information is held in STM—can also determine what is attended to amongst preattentive information (Wolfe and Utochkin, 2019).

Most references to preattention are concerned with visual information. But other non-focus exogenous (sensory) information is within the scope of preattention, as may be endogenous information concerning one’s physiological or emotional state, or other matters that are not the current focus of attention.

The hypothesis proposed here is that WM and attention evolved before the evolution of brains, and have been inherited by vertebrates and arthropods since their last common ancestor (LCA). And, because molluscs and nematodes evolved after the divergence of vertebrates from the predecessors of arthropods, early molluscs and nematodes would probably have inherited WM and attention, though some may have subsequently lost these functions, as may some vertebrates and arthropods.

This report is in two parts. The first concerns the nature, relationship and neural location of WM and attention in humans. It briefly lists some properties of WM and attention in humans, and some interactions between WM and attention, as determined from cognitive behavioural studies and neuroscientific experiments.

The second is a collation of evidence that WM and attention are likely very ancient. It summarises WM and attention data on four phylogenetically diverse species for which there is detailed published evidence concerning the properties of WM and attention possessed by them. This is extended by reports of WM in many other species from related clades, plus various supplemental evidence. The data and arguments are presented in five stages:

1. Data concerning archerfish, and other vertebrate classes, which indicate that WM was very likely possessed by early vertebrates.
2. Data concerning jumping spiders, honey bees, and other arthropods, providing some evidence for possession of WM by early arthropods.
3. WM in *Octopus vulgaris*, some other molluscs, and possibly *Caenorhabditis elegans*, a nematode.
4. The phylogenetic relationship between these clades.
5. Claims that the tripartite brain was inherited from the LCA of vertebrates and arthropods.

## 2. Working memory and attention in humans

### 2.1. WM and attention are forms of information

WM is information stored for a period of time in a state that makes it immediately available for use by brain processes that determine responses. Thus, WM is stored information of a particular kind.

The standard view of attention is that it is enhancement of focus information, and inhibition of non-focus information. But this is not strictly correct. Attention is not a process; it is an information state. What we experience as attention is a more detailed, or clearer, experience (increased information density) at the focus, accompanied by a less detailed, less clear, experience (reduced information density) outside of the focus. Thus, attention is a specific kind of localised variation in information density.

WM and attention are both information of particular kinds that can influence behaviour. This is because WM and attention, which are outputs from certain brain processes, are also inputs to other brain processes that determine behaviour.

### 2.2. Working memory in humans

The current view is that WM is information which is retained by an organism for a short time after an event, generally for seconds or minutes (Smith and Jonides, 1999), that is available for use in the selection, storage, and activation of responses (Awh and Jonides, 2001; Cowan, 2008; Oberauer, 2019). In some situations, such as the activation of a plan, or having an intention to do something, WM may retain information for a longer time. WM enables a wide range of activities. When there is no longer incoming information available to an animal about a continuing potential threat or opportunity, it depends upon information in WM to prepare its response. WM can also enhance the ability of its possessor to identify objects in its environment and their movements by allowing incoming information to be compared with WM-stored information, as objects of interest change their position. WM is the temporary activation of focal information, STM, or LTM (Fuster, 1997).

Humans have WM for many, possibly all, sensory modalities, plus WM for other important information, such as intended actions (Miller et al., 1960; D’Esposito and Postle, 2015). There is published evidence for:

- *auditory* WM (Samms et al., 1993; Awh et al., 1996; Maybery et al., 2009; Kaiser, 2015; Linke and Cusack, 2015; Kumar et al., 2016);
- *olfactory* WM (Herz and Engen, 1996; White et al., 1998; Andrade and Donaldson, 2007; Zelano et al., 2009);
- *gustatory* WM (Lara et al., 2009; Daniel and Katz, 2018);
- *haptic* WM (Sullivan and Turvey, 1974; Harris et al., 2002; Reed et al., 2005); and
- *visual* WM. Most research has been with visual WM, and all of the research on WM in humans discussed below concerns visual WM. Similarly, the discussions of attention refer to visual attention, except where indicated.

### 2.3. Attention in humans

#### 2.3.1. Whatever is important, novel, or salient tends to determine the focus of attention

Attention in humans tends to be drawn to information that is, or could be, biologically, socially or personally important. Attention is preferentially captured by bodies and faces (Downing et al., 2004; Ro et al., 2007; Salvato et al., 2017), particularly faces turned toward the observer (Stein et al., 2011; Chen and Yeh, 2012; Gobbini et al., 2013a). Attention tends to be drawn to personally important matters, such as familiar faces (Gobbini et al., 2013b) or one’s own name (Moray, 1959; Wood and Cowan, 1995).

Attention is also attracted to fear-related objects more quickly than objects that are unlikely to provoke fear (Ohman et al., 2001; Lin et al., 2009). Animate objects capture attention more readily than inanimate objects (New et al., 2007; Calvillo and Hawkins, 2016; Saryazdi et al., 2019). Attention tends to be rapidly captured by motion, especially the onset or cessation of movement, or by looming objects (Hillstrom and Yantis, 1994; Abrams and Christ, 2003; Franconeri and Simons, 2003; Kawahara et al., 2012; Smith and Abrams, 2018). Each of these are preferentially attended to because they may be biologically or socially important to the individual.

Attention tends to be captured by salient visual stimuli (Nothdurft, 2002; Forster and Lavie, 2008; Geng and Diquattro, 2010; Kerzel and Schönhammer, 2013; Gayet et al., 2014) or unexpected sounds (Cherry, 1953). Reviews of visual salience in humans (Wolfe and Horowitz, 2004, 2017), concluded that colour, motion, orientation and size are attributes of objects that commonly lead to “pop-out”. Under some conditions, salient stimuli do not capture attention (Yantis and Jonides, 1990), and this has been explained on the basis that “attend-to-me” signals can be overridden by top-down suppression to prevent the capture of attention (Sawaki and Luck, 2010; Gaspelin and Luck, 2018).

#### 2.3.2. Attention is enhanced focus information and reduced non-focus information

Attention is enhanced information concerning its current focus, accompanied by reduced information concerning matters that are irrelevant to the focus. This has been demonstrated in behavioural experiments (Posner, 1994; Awh and Jonides, 2001; Awh et al., 2006; Soto et al., 2008; Cohen et al., 2012; Bahmani et al., 2019), and by noting neural responses to focus-relevant and focus-irrelevant information (Pinsk et al., 2004; Yi et al., 2004; Schwartz et al., 2005; Torralbo et al., 2016).

Under attentional load the enhancement of focal information and reduction in non-focal information are increased (Lavie and Tsal, 1994; Lavie, 1995; Rees et al., 1997; Pinsk et al., 2004; Yi et al., 2004; Schwartz et al., 2005). “Attentional load” is the difficulty, or importance, of identifying or tracking an object of interest. When attentional load is greatest, the information from the attentional focus—which may involve any sensory modality—is maximised, and irrelevant information is minimised, whether from the same or a different sensory modality (Molloy et al., 2015). Attentional load is commonly achieved in experiments by making the object of attention difficult to identify. It is likely that attentional effects on incoming information are graduated according to the attentional load (Torralbo et al., 2016).

Enhanced information about the focus increases the probability of correctly identifying and monitoring objects. This would be very adaptive, and leads to an expectation that attention could have been a very early evolutionary development in mobile animals. Attentional processes that inhibit information which is irrelevant to the focus reduce the possibility that attention—and WM—will move away from the matter currently of interest.

### 2.4. The relationship between WM and attention

It is widely accepted that, in humans, working memory and attention are closely related (Awh and Jonides, 2001; Berti and Schröger, 2003; Oberauer, 2019):

1. Attention is enhanced focal information and reduced non-focal information, as noted above, particularly under high attentional load, when a difficult-to-identify, or important matter, is in focus (for example, Lavie, 2005).
2. When attention moves to a new focus, information tends to be lost (Hayhoe et al., 2002).
3. The problem of losing important information when attention moves is overcome by information concerning the focus being held in STM or WM.
4. Subsequent to the transfer of focus-related information to WM, processes operating with WM information tend to maintain the focus of attention, thereby reducing unwanted movement of attention away from the focus (Lavie and de Fockert, 2005; Awh et al., 2006; Soto et al., 2008; Cohen et al., 2012; Dowd and Mitroff, 2013; Williams et al., 2013; Olivers and Roelfsema, 2020).
5. Attention may continue to collect focus information, which is transferred to WM (Soto and Humphreys, 2007; Soto et al., 2008; Dalton et al., 2009; Ko and Seiffert, 2009; Zhang et al., 2018).
6. The persistence of attention to a focus stabilises WM (Smyth and Scholey, 1994; Awh and Jonides, 2001; Matsukura et al., 2007; D’Esposito and Postle, 2015).
7. Brain processes operate with incoming focal information and information held in WM to determine a response.
8. If a response—an “action plan”—is selected but not immediately activated, it is copied into STM or WM (Miller et al., 1960; Oberauer, 2019), and attention may be focused in such a way as to prevent the information from being over-written—generally, the focus is appropriate to the first action of the sequence in the action-plan (Miller et al., 1960; Awh and Jonides, 2001), when the first action is completed, attention, and WM move to the next action in the sequence (Hayhoe et al., 2002).
9. When attention activates information in STM or LTM, this information is immediately available for use by brain processes. Processes that determine the focus of attention may also be influenced by this active information, and therefore tend to maintain the focus on information already in WM.

### 2.5. The location of WM and attention

Since the 1990s, evidence has accumulated that WM and attention are located in relevant sensory areas of the brain. Much of the early evidence is summarised in Fuster (1997) who reported that short-term memory (STM) and long-term memory (LTM) appear be stored in the same location. He reported that WM is temporary activation—increased firing of neurons—of STM or LTM; a view supported by other researchers (Kastner and Ungerleider, 2000; Awh and Jonides, 2001; Barnes et al., 2001; Jonides et al., 2005; Postle, 2006; Lewis-Peacock and Postle, 2008; D’Esposito and Postle, 2015). Fuster claimed that almost all regions of the brain store information of some kind, and that information is held with some of the neural systems that manipulate it, and this is now widely accepted (for example, Jonides et al., 2005; D’Esposito and Postle, 2015).

Processes associated with visual attention and visual WM are located in the visual cortex. Therefore, it is possible that attentional selection and storage of information, together with some associated processes may share common neural mechanisms and circuits, and that these complexes of information storage and associated mechanisms probably occur in each sensory information store (Kastner and Ungerleider, 2000).

A number of experiments employing multivariate pattern analysis (MVPA) with fMRI of early visual areas have determined the location of WM information. Using MVPA with fMRI of the V1 region of the human visual cortex, Haynes and Rees (2005) were able to determine which of two stimuli were being viewed by participants even when the stimuli were masked, and invisible to the participants. Similarly, Kamitani and Tong (2005) found that fMRI signals in early visual areas could predict which of eight stimulus orientations a subject was focussed on. When subjects had to attend to one of two overlapping orthogonal gratings, activity was biased toward neural structures representing the orientation attended to (Kamitani and Tong, 2005). These results are evidence that visual attention and WM occur in early visual areas, and that WM is associated with the activation of neural structures.

In MVPA fMRI analyses, Serences et al. (2009) also reported that the maintenance of information in visual WM is associated with activation patterns in the regions of the visual cortex that encode the sensory information. But, Harrison and Tong (2009) found that early visual areas V1 to V4 can retain information about visual features over periods of many seconds with little or no sustained activity. This may be an attentional-load effect; Offen et al. (2009) reported that the early visual cortex exhibited sustained responses throughout the delay period when subjects performed attention-demanding tasks, but the delay-period activity was not distinguishable from zero when subjects performed a low attentional load task. It is possible that in the low attentional load task, the relevant information was held in short term memory, which could be retained with little or no neural activity, but in the higher load task the memory was held in an active state.

In two fMRI studies; one a delayed-recognition memory for location versus identity of abstract geometric shapes (Postle and D’Esposito, 1999); and the other of “n-back”, in which a sequence of shapes is presented and the subject has to strike a key in response to a shape that they previously saw either 2 or 3 stimuli earlier (Postle et al., 2000); both found object-specific WM-related activity in the ventral temporal and occipital cortex regions associated with the relevant sensory processing. Again, sensory WM was being stored in relevant sensory areas, and was detectable under these high-load conditions.

There has been recent discussion concerning the neural state of WM, with various reports that WM information may be represented in an “active or silent state”, or in “active or passive storage” (Mongillo et al., 2008; Manohar et al., 2019; Stokes et al., 2020). However, these reports do not conflict with the view that WM is generated by attending to information held in relevant brain areas.

It is important to note that WM in humans is not necessarily conscious (Hassin et al., 2009; Soto et al., 2011; Bona et al., 2013; Soto and Silvanto, 2014, 2016; Bergström and Eriksson, 2015, 2018; Trübutschek et al., 2017). There is also evidence that attention is not necessarily conscious (Kentridge et al., 1999, 2004; Koch and Tsuchiya, 2007; van Boxtel et al., 2010; Chong et al., 2014; Bergström and Eriksson, 2015; Prasad and Mishra, 2019). Therefore, it is not necessary for species to possess consciousness in order for them to have WM or attention. Also, evidence that species possess WM or attention tells us nothing about whether those species possess consciousness.

In summary, research concerning the neural location of WM and attention indicate that:

1. STM, like LTM, is based on synaptic strength, not ongoing neural activity.
2. STM and LTM, and attentional selection, occur in the relevant information store, together with the mechanisms that generate them, and some of the mechanisms that utilise them.
3. Attention to incoming sensory information, or to information represented in STM or LTM, generates WM.
4. Storage (as STM or LTM) and attentional activation as WM are systems that do not require consciousness, and may not require a complex brain, and therefore it may be possible for animals with relatively simple nervous systems to possess WM.

It is noteworthy that honey bee visual WM is also located in the early visual areas of the bee brain, and is activated by attention to a task (Paulk et al., 2014), just as with humans.

## 3. The evolution of WM, and its possible loss in some species

The evolution of WM may not have been a very complex development on the scale of evolutionary changes over time. There appears to be no evidence concerning how WM evolved, but it could have evolved from stimulus–response (S-R) actions, which are possessed by organisms with very simple nervous systems (Reber, 1995). S-R actions, in their simplest form, are fixed behaviours in response to specific stimuli. An example would be when an animal senses the presence of a predator, and either freezes, moves away very rapidly, or withdraws immediately into a safe place.

It is likely that attention evolved early on, so as to enhance S-R behaviour. This is because attention would have permitted improved detection of some types of stimulus in S-R events.

In general, organisms that possess S-R behaviour respond differently to successive similar S-R events. Relatively strong S-R type responses that do not lead to a significant positive or negative outcomes subsequently diminish in strength (they exhibit “inhibition”); and relatively weak S-R type responses that lead to significant outcomes are subsequently enhanced (“sensitisation”). These are the simplest forms of behavioural adaptation in multicellular mobile animals (Roth, 2013).

Inhibition and sensitisation require temporary storage of information concerning the nature and outcomes of each S-R event, and some processing of this information in response to the next similar S-R event. Thus, prior to the evolution of WM there would have been storage of relevant data and processing of this data. It would seem a relatively small change, in evolutionary terms, for these forms of information storage and processing to become STM, that could be activated by attention as WM and processed to generate a response.

These comments are not proposing a hypothesis for the evolution of WM. They merely demonstrate that the acquisition of WM could have been a fairly simple, but adaptive, evolutionary change.

For most species, WM is very adaptive. It permits flexibility of responding by allowing details of the situation to be incorporated into the choice of response. Ultimately, all mental processes from remembering something no longer sensed, or knowing the way home; to problem-solving, thinking, and planning; requires that relevant information be held in STM and activated by attention for processing (Roth, 2013; Hahn and Rose, 2020; Buschman, 2021). The WM mechanism is a basic component of mind^1^ that is necessary for all response systems (other than S-R mechanisms) to function. This would make WM and attention, core mechanisms upon which all further development of mind depended (D’Esposito and Postle, 2015).

The evolution of WM was a very significant event in the early history of mobile animals. Nevertheless, many species have continued to thrive without WM, and it is possible that some species, whose forebears possessed WM, could have lost WM if their lifestyle changed so that they no longer needed its functionality.

If a species has lost WM due to evolutionary changes, it would be expected that its lifestyle would explain why that occurred—why S-R behaviour is all that is required for continuance of the species. In general, this would require that sufficient of these animals are able to obtain food and avoid predators until they have produced offspring. This is likely to involve the production of many offspring, and not having to take special action to find food, avoid predators, or find a mate. Examples would be living on or in a food source, or reflexively catching passing food in their environment; having evolved protections against predation; and finding mates by responding directly to sensory cues.

Any function can be lost through genetic changes if the changes are not selected against in the situation of the given organism. Or a function may be lost during one or more of its life stages if the loss is not selected against. For example the Mexican cave fish (*Astyanax mexicanus*) lost its sight as a result of living in darkness. Deleterious changes in eye development genes were not selected against, and may have been selected for in the lightless environment (Torres-Paz et al., 2018). If a function is no longer essential to survival of the gene pool, mechanisms supporting that function may be selected against when a suitable genetic change occurs, because it is more efficient use of resources not to develop mechanisms that are no longer needed.

## 4. WM and attention in archerfish and other vertebrates

The second part of this report summarises published WM and attention data for a range of non-human species for comparison with human WM and attention, and as evidence for the early evolution of WM and attention. We begin with another vertebrate; archerfish.

### 4.1. WM and attention in archerfish

Archerfish (*Toxotes* sp.) live in small groups in mangrove swamps and brackish or fresh water in rivers and estuaries (Lüling, 1963). They are noteworthy for downing prey—flies, spiders or even small lizards—from overhanging vegetation with precisely aimed shots of water (Bekoff and Dorr, 1976; Schuster, 2007), and can even hit moving targets (Schuster et al., 2006; Ben-Simon et al., 2012).

In its native habitat the archerfish swims at the surface or just beneath it, the great eyes peering upward in what appears to be a purposeful search for prey. When it spots a likely insect on a water plant or a mangrove root, it takes up a characteristic position with its snout just breaking the surface (Lüling, 1963, p. 100).

Upon reaching the water surface the fish (*T. chatareus*) remains motionless for a few seconds at a fixed body angle. During this period the eyes may be seen to rotate in the dorsoventral plane and to converge. This binocular fixation of the prey would allow its correct placement along the extension of the fish's longitudinal axis. It may also allow the archerfish to judge the prey's distance (Dill, 1977, p. 173).

When archerfish have searched in a particular area without success they tend not to look there again for some time (Gabay et al., 2013). This is referred to as “inhibition of return”, and is a valuable ability for foragers because it makes foraging more efficient. Inhibition of return requires the possession of a WM to retain a record of recent locations, and it indicates that archerfish possess WM.

After being trained to shoot at targets on a monitor screen, archerfish demonstrated “expectation of location” and inhibition of return in an experiment in which an on-screen red or green mark indicated which side of the screen a target would appear (and provide a reward if hit). Fish demonstrated expectation of location by responding to the target more quickly when it was correctly positioned than when it was incorrectly positioned. When the target appeared after a delay, the fish took longer responding to the correctly positioned target than to the incorrectly positioned target. After initially not finding the target in the correct location, archerfish were slower finding targets due to a tendency not to look in the same place twice—inhibition of return (Saban et al., 2017). Expectation of location and inhibition of return both require a WM.

An archerfish (*T. jaculatrix*) can predict the point where the prey, when dislodged by a water jet, will land on the water surface. When one of their shots dislodges an insect, all the fish in a group are able, within 100ms, to begin turning to align their body axes to the spot where the insect will hit the water surface (Rossel et al., 2002). The shooter and other members of its group rapidly turn toward the prey impact direction and head toward it at a speed that is adjusted to arrive about 50ms after the prey hits the water. Archerfish can predict the direction and the distance to the point of impact of dislodged prey, and use this ability to determine their direction and speed of movement (Wöhl and Schuster, 2006). This requires WM for the calculation and for retention of their action plan.

Archerfish (*T. jaculatrix*) shooting at prey vary the velocity of parts of the jet so that the faster-moving tail catches up to the head of the jet just prior to impact with the prey, and a single water drop hits the prey with a large momentum (Vailati et al., 2012; Gerullis and Schuster, 2014). This momentum is sufficient to overcome the strong anchoring forces of the insect (or other) prey. When shooting at insect, spider, or lizard prey, archerfish match the impact momentum of shots to the strength of prey adhesion (Schlegel et al., 2006). For this to occur, there would need to be complex calculations, based upon innate mechanisms, which would require retention of various data in a WM.

In different experimental arrangements, archerfish (*T. chatareus*) can exhibit serial or parallel visual search (Rischawy and Schuster, 2013; Ben-Tov et al., 2015; Reichenthal et al., 2019). Evidence of parallel search demonstrates that archerfish respond to preattentional information.

In summary, there are four lines of published evidence that archerfish (*Toxotes* sp.) possess WM:

1. By displaying inhibition of return.
2. By displaying expectation of location.
3. By virtue of being able to hold data sufficiently to carry out complex calculations to determine the direction and distance to the point of impact of falling prey, and to adjust their speed of movement so as to arrive just after the prey lands on the surface of the water.
4. By making adjustments to the water jet to allow for prey size.

It is noteworthy that archerfish (*Toxotes* sp.), which amongst vertebrates are phylogenetically very distant from humans, possess WM and attentional responses that are similar to humans, though the LCA of archerfish and humans lived about 400mya (Erwin et al., 2011; Briscoe and Ragsdale, 2019). This suggests that WM and attention in vertebrates may have been conserved over a very long period.

### 4.2. WM in other vertebrates

There are reports of WM in very many vertebrate species, especially among mammals and birds, but there is less published evidence of WM in fish, amphibians and reptiles. In Table 1, I have listed some WM-possessing species from five vertebrate classes: fish, amphibians, reptiles, mammals, and birds, which demonstrates the widespread possession of WM amongst vertebrates.

**Table 1:** WM in five vertebrate classes.

Class	Species		Evidence of WM	References
Fish	Rock pool goby	*Bathygobius cocosensis,* *B. krefftii*	Remembered the deepest pool	White and Brown, 2014
	Red sea clown fish	*Amphiprion bicinctus*	Took direct route home	Bshary et al., 2002
	Nine-spined stickleback	*Pungitius pungitius*	Proportional observation learning	Pike et al., 2010
Amphibians	Newt	*Taricha torosa*	Path integration	Endler, 1970
	Frog	*Allobates femoralis*	Successful in a detour task	Munteanu et al., 2016
Reptiles	Tortoise	*Geochelone carbonaria*	Flexible observational learning	Wilkinson et al., 2010
	Arboreal lizard	*Anolis evermanni*	Solved a lid-opening problem	Leal and Powell, 2011
Mammals	Bottlenose dolphin	*Tursiops truncatus*	Invented an action plan	Kuczaj et al., 2010
	Hyena	*Crocuta crocuta*	Solved a problem	Benson-Amram and Holekamp, 2012
Birds	Rook	*Corvus frugilegus*	Solved problems by using tools	Bird and Emery, 2009
	Chickens	*Gallus gallus*	Chicks knew where most “friends” went	Rugani et al., 2011
	Pigeon	*Columbia livia*	Chose future route	Gibson et al., 2012

**Table 2:** Some arthropods with WM.

Clade	Species		Evidence of WM	References
Spiders (Chelicerates)	Western black widow	*Latrodectus hesperus*	Path integration	Sergi et al., 2021
	Golden orb-web spider	*Nephila clavipes*	Retained memory of number and size of prey	Rodríguez et al., 2015
	Wolf spider	*Lycosa tarantula*	Path integration	Reyes-Alcubilla et al., 2009
Scorpion (Chelicerate)	Lesser Asian scorpion	*Mesobuthus eupeus*	Path Integration	Prévost and Stemme, 2020
Insects (Mandibulates)	Bull ant	*Myrmecia midas*	Compared current scene to a memorised scene	Freas et al., 2018
	Fruit fly	*Drosophila melanogaster*	Remembered position of target when not seen	Neuser et al., 2008
	Dung beetle	*Scarabaeus galenus*	Path integration	Dacke et al., 2020
Crustaceans (Mandibulates)	Fiddler crabs	*Uca rapax,* Various species	Path integration Path integration	Murakami et al., 2017; Zeil and Layne, 2002

There are many ways one can determine whether an animal possesses a WM. The lines of evidence that I have chosen are: path integration, remembering the way to a safe place, observational learning (that cannot be ascribed to associative learning), problem solving, and developing and retaining a plan of action (such as for a detour task, or any complex route).

**Figure 1:**
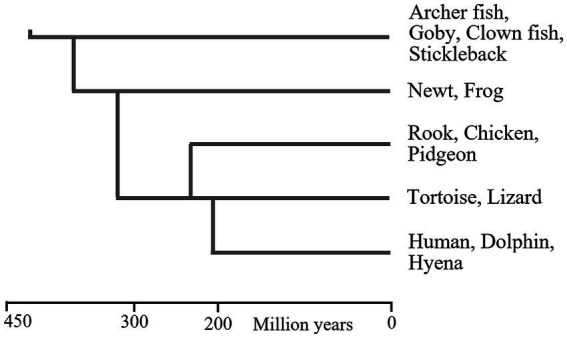
Phylogenetic relationships between vertebrate classes. This figure provides a general impression of the dates at which these animal classes first evolved. There are various problems-associated with fossil identification and molecular clocks-that can result in large differences between reported dates (Cunningham et al., 2016). Approximate dates of the LCA of pairs vertebrate classes (shown as vertical lines) were extracted from figures in Laurin and Reisz (1995), Kemp (2005), Alcober, et al. (2010), Godefroit et al. (2013), Nesbitt et al. (2013), Marsicano, et al. (2016), Zhao et al. (2021).

The phylogenetic relationships between these vertebrate classes are shown in Figure 1, and indicate that WM may have existed since the early evolution of bony fish, about 420mya. It is possible that WM separately evolved in each of these vertebrate classes, but this is unlikely, because they all have comparable brain structures and functionalities (Karten, 2015; Murakami, 2017; Pessoa et al., 2019), which implies that all have some cognitive abilities that would require WM.

Some cartilaginous fish, which predated bony fish, may also have WM. Manta rays and some sharks have high brain/body ratios but it is very difficult to observe their behaviour or ascertain their abilities (Ari, 2011), but manta rays do exhibit cognitive responses (Ari and D’Agostino, 2016). The earliest cartilaginous fish predated bony fish by about 40 million years (Benton, 2005).

Cyclostomes, which evolved earlier than cartilaginous fish, and of which only lamprey and hagfish survive, may also possess WM. Suryanarayana et al. (2017, p. 12) wrote that,

the basic features of the mammalian cortices had already evolved when the lamprey line of evolution diverged from that leading up to mammals some 500 million years ago. It would seem that this basic organization evolved to control the limited behavioral repertoire of the lamprey and that it has been maintained albeit expanded during evolution to control the more complex and refined movements and sensory processing of ‘higher’ vertebrates.

This suggests that the lamprey and hagfish—which have comparable brain structures to each other (Dupret et al., 2014), and comparable brain structures to vertebrates (Pani et al., 2012; Murakami, 2017)—have some cognitive flexibility, for which a WM would be required. Lamprey and hagfish first evolved about 500mya (Kuraku and Kuratani, 2006; Smith et al., 2010; Shimeld and Donoghue, 2012).

According to Sugahara et al. (2016, 2017), the brain regionalisation seen in gnathostomes (vertebrates possessing jaws) dates back to before the divergence of jawless fish and jawed fish. This would mean that some form of cognition, necessitating the possession of WM, existed in very early vertebrates. Apparently, there has been some subsequent degeneration of brain structures in hagfish (Sugahara et al., 2017).

In conclusion, it is likely that some members of all vertebrate classes possess WM, which suggests that WM existed at the time of the LCA of vertebrates—the most recent species or gene pool that was ancestral to all vertebrates. (But some vertebrate species may have since lost WM.)

## 5. WM and attention in salticids, bees and other arthropods

Next, we look at evidence for WM and attention in arthropods. We begin with jumping spiders because there are very many published reports of relevant behaviour in these species.

### 5.1. WM and attention in salticids

A common test for WM in animals is to interrupt sensory input concerning an object of interest for a short time, and observe their behaviour after the sensory input is re-established. In separate tests, sensory input is re-established with the object unchanged (the control condition), or with the object changed, and noting whether animals behave differently under the two conditions, indicating “expectancy violation”, which constitutes evidence of WM.

Cross and Jackson (2014) used an araneophagic (spider-eating) salticid, *Portia africana*, in expectancy violation tests for working memory. Their experiments began with a test spider on a ramp facing a lure (dead prey-spider mounted on a cork disk) that could be reached by jumping. After the test spider faced the lure for 30s, its view of the lure was blocked by lowering an opaque shutter. When the shutter was raised 90s later, either the same lure came into view again (control) or a different lure came into view (experimental). Cross and Jackson reported that when prey type was changed, attack frequency was significantly lower than in control trials when the prey was unchanged, and this demonstrated that *P. Africana* possesses a visual WM.

#### 5.1.1. Salticids can hold an action plan in WM

Another means to determine the presence and properties of WM is to observe whether an animal is able to continue a sequence of actions when the goal of those actions is no longer sensed. Fifteen species of araneophagic salticids were tested in an experimental arrangement with two raised metal pathways, each with multiple changes of direction (Cross and Jackson, 2016). The spiders began each trial on top of a tower from which they could view the two pathways, with a prey box at the far end of each; one path had a dead prey-spider in the box, but the other path led to an empty box. From the top of the tower, the spiders could see the two routes, and they appeared to make a thorough visual examination of the scene. To reach the paths, the spiders had first to descend the tower to the base on which it and the paths were mounted, from where they could no longer see or smell the prey.

The great majority of the spiders chose the correct route; the number of correct choices was statistically significant for all 15 species. It appeared that each of these species of salticids retained a working memory of how to get to the prey spider. When descending the tower, the spiders seemed intent on the start of the correct path, which is a common phenomenon with WM action plans.

Describing another detour experiment with the salticid, *P. fimbriata*, Tarsitano (2006, p. 1437) wrote that “*Portia*… plans the initial stage of its detour by scanning the possible route and picking out an unbroken path from start to goal and then aiming at an initial objective along the detour”. This is typical of situations where an action plan is retained in WM. The first important stage in such an action plan would be the start of the correct pathway, and attention on this point would tend to prevent loss of the action plan from WM (Hayhoe et al., 1999; Land et al., 1999; Land, 2006; Schütz et al., 2011).

#### 5.1.2. Salticid retains knowledge of the number of prey seen and the path to them

The salticid, *P. Africana*, is able to retain in WM some limited knowledge of the number of prey that it has seen and how to get to them (Cross and Jackson, 2017). *Portia* saw between one and six spiders at the start of the experiment, but during movement towards the prey its view of the prey was obscured for about one minute, and the number of prey was changed during that time. When the prey were again in sight, a significant number of spiders did not continue towards the prey after certain changes in the number of prey. The results indicated that *Portia* had recorded in STM that there were 1, 2, or ‘many’ prey, and retained this information for the period of one minute that the prey were out of sight. Apparently, when *Portia* saw the prey again, its attention was drawn to the number of prey recorded in STM, which became its WM, and caused expectancy violation after certain changes in the number of prey.

#### 5.1.3. Salticid has visual and olfactory WM and exhibits attentional load effects

*Evarcha culicivora* is a specialist salticid that feeds indirectly on vertebrate blood by eating mosquitos (Cross and Jackson, 2010a). *E. culicivora* uses sight or smell to select as prey a female mosquito that has taken a recent blood meal. Cross and Jackson (2010a,b) primed *E. culicivora* with the sight or smell of either blood-carrying mosquitoes or potential mates, and found that these salticids showed evidence of WM for visual or olfactory information. They also exhibited an attentional load effect for both sensory modalities—when visual or olfactory information relevant to WM was difficult to detect, other important sensory information was not noticed.

#### 5.1.4. Salticid secondary eyes appear to have a preattentional function

Salticids have four pairs of eyes, the principal, forward-facing eyes with high visual acuity, and three pairs of lower acuity eyes; the anterior lateral (AL), posterior medial, and posterior lateral (PL) eyes (Harland et al., 2012). Evidence is accumulating that the AL eyes, and probably the PL eyes, have a preattentional function, and that whilst the principal eyes are focused on an object, information from the secondary eyes is analysed and may result in the focus of the principal eyes changing (Land, 1971, 1972), or in the preparation of some other response.

Various experiments have demonstrated that images or objects presented to the AL eyes can result in redirection of the principal eyes towards the object (*Servaea vestita*: Zurek et al., 2010; Zurek and Nelson, 2012; *Phidippus audax*: Jakob et al., 2018; *Menemerus semilimbatus*: De Agrò et al., 2021). Salticids do not turn toward all moving targets in the visual field, which suggests selective attention (De Agrò et al., 2021).

Looming images presented to the AL eyes mediated a response by the principal eyes, but shrinking images did not (*P. audax*: Spano et al., 2012; Bruce et al., 2021). Movement of the principal eyes towards a second image was significantly less likely when the spiders were already looking at an image of prey (a cockroach) than when they were looking at a blank screen or an oval shape (*P. audax*: Bruce et al., 2021). Apparently, the redirection of the gaze of the principal eyes from a primary stimulus to a new stimulus detected by the AL eyes is flexible, and depends on characteristics of both the primary and the distractor stimuli.

Together the AL eyes and PL eyes provide almost a 360° field of view (Harland et al., 2012). The salticids appear to exercise a form of preattention, *via* their AL eyes, and probably also their PL eyes, which appear to have similar functions (De Agrò et al., 2021).

#### 5.1.5. Summary of WM and attention data for Salticids

The various experiments investigating WM and attention in salticids were not confined to a single species unlike the experiments with humans. However, the range of salticid species demonstrating similar behaviours in these experiments would seem to make it reasonable to collate the data for the different species and assume that, for at least some salticids, the collated results would apply. The salticid data may be summarised as follows:

- Salticids possess WM (Tarsitano and Jackson, 1997; Tarsitano, 2006; Cross and Jackson, 2010a,b, 2014, 2016).
- Salticids possess *visual* and *olfactory* WM (Cross and Jackson, 2010a,b).
- WM in salticids is generated by immediately prior sensory information that is attended to (Cross and Jackson, 2010a,b, 2014).
- Under attentional load, incoming information relating to the content of WM is enhanced, whilst incoming information unrelated to the content of WM is diminished (Cross and Jackson, 2010a,b).
- Action plans are stored in WM (Tarsitano and Jackson, 1997; Tarsitano, 2006; Cross and Jackson, 2016), and retained in WM by attentional mechanisms.
- Salticids appear to have a preattention mechanism based on information from their secondary eyes.

Subject to the limitations of the published information on salticids, it appears that the properties of WM and attention in salticids and humans are very similar. This could occur either because an attention-WM mechanism with similar properties separately evolved in the ancestors of salticids and humans, or because salticids and humans inherited this mechanism from a common ancestor.

### 5.2. WM and attention in honey bees (*Apis mellifera*)

Honey bees (*Apis mellifera*) belong to a different large clade of arthropods from that of salticids. Bees are mandibulates; spiders are chelicerates. These two clades are believed to have diverged about 550mya, so bees and spiders are phylogenetically very distant from one another. Similarities between WM and attention in species from these two clades contributes some evidence for their presence in the LCA of arthropods.

#### 5.2.1. WM in honey bees

In an unfamiliar environment, bees memorise landmarks as they move away from their starting place, and use these to return (Gould, 1988). After feeding, bees memorise the landmark panorama around the feeding place: “every bee passively transported to a novel feeding site and allowed to perform a TBL [turn back and look] there returns to that site, while bees prevented from performing a TBL never come back” (Lehrer, 1991, p. 273). Their ability to return after the TBL requires a WM.

In familiar territory, honey bees navigate according to a spatial memory of some kind. Bees were captured en route to a food source or when returning to the hive, and were released at a different location. After reorienting themselves, they continued to their original target—the feeding station or the hive (Menzel et al., 2005). These findings suggest that, depending on their circumstances, honey bees exhibited information WM (map-like memory), and an action plan in WM (for continuing their prior task).

A recent view (Hoinville and Wehner, 2018; Kheradmand and Nieh, 2019; Even et al., 2020) regarding bee navigation, is that recorded aspects of the environment are combined with path integration and other incoming sensory information by bees to find the way to the nest, which would require WM.

The honey bee “dance”, performed by bees upon returning to the colony after successfully locating food, provides nest-mates with information on the quality, direction, and distance of the food source (Von Frisch, 1967; Riley et al., 2005; Grüter and Farina, 2009). All of this information would have to be stored and used to determine the future movements of the “recruited” foragers—to generate their action plan—and would require a WM. Additionally, the bees collect odour cues from the successful forager and store this odour information for locating the food source when they are close to it (Von Frisch, 1967; Farina et al., 2005).

There are many published laboratory experiments demonstrating that honey bees retain information in WM. As examples, Zhang et al. (2005) reported that bees can retain a simple visual pattern in WM for up to 5s, and Howard et al. (2019) reported that bees can retain a colour signal in WM and use it, after a delay, to correctly carry out a task to “add one” or “subtract one”, as trained to do in response to the colour.

#### 5.2.2. Attention in honey bees

Various experimental techniques have demonstrated that bees exhibit attention (Zhang and Srinivasan, 1994; Spaethe et al., 2006; Morawetz and Spaethe, 2012; Paulk et al., 2014; Avarguès-Weber et al., 2015). Paulk et al. (2014) recorded brain activity in tethered honey bees, which by walking on an air-supported ball could move an on-screen image of a vertical green bar to a position in front of their eyes. When they did so, attention to the bar was accompanied by activity in the early visual areas of the bees’ brains, suggesting that processes associated with attention were located in these regions of their brain.

#### 5.2.3. Preattention in honey bees

There have been conflicting reports on whether *A. mellifera* exhibits evidence of preattention in visual search tasks. Spaethe and co-workers (Spaethe et al., 2006; Morawetz and Spaethe, 2012) reported that bees perform serial searches, but other researchers found evidence for preattention processing in bee behaviour (Zhang and Srinivasan, 1994; Dyer et al., 2005; Avarguès-Weber et al., 2015).

#### 5.2.4. Summary of WM and attention data for honey bees

Observations and experiments with honey bees (*A. mellifera*) have shown that:

- Honey bees possess WM.
- Bees possess *visual* and *olfactory* WM.
- Action plans are stored in honey bee WM.
- Visual attention mechanisms are located in the early visual areas of bees’ brains.
- Bees appear to possess preattentional processing that is active in some circumstances.

Subject to the limitations of the published information, it appears that, as with salticids, the properties of WM and attention in honey bees and humans are very similar. This could be because bees and humans both inherited this mechanism from a common ancestor. But it is possible that an attention-WM mechanism with similar properties separately evolved in the ancestors of bees and humans. The fact that humans, archerfish, salticids and honey bees have WM with very similar properties (see Table 3) together with evidence of WM in other vertebrate classes and other chelicerate and mandibulate arthropods suggests that they may have inherited WM from a common ancestor.

**Table 3:** Summary of data for two deuterostomes (D) and four protostomes (P).

Species	Clade	D or P	WM	Action plan in WM	Attention	Preattention	Attentional load effect
Humans	Mammal	D	Yes	Yes	Yes	Yes	Yes
*Toxotes* sp.	Teleost	D	Yes	Likely	Yes	Yes	No data
*Salticid* sp.	Chelicerate	P	Yes	Yes	Yes	Yes	Yes
*A. mellifera*	Mandibulate	P	Yes	Yes	Yes	Likely	No data
*O. vulgaris*	Mollusc	P	Yes	Yes	Yes	No data	No data
*C. elegans*	Nematode	P	Likely	Likely	Likely	No data	No data

### 5.3. WM in other arthropods

Many other arthropods have been observed to possess WM; a small selection of them is shown in Table 2. As noted earlier, salticids and honey bees belong to two different clades of arthropods; salticids are chelicerates, and bees are mandibulates. Some other chelicerates and mandibulates are included in Table 2.

In Table 2, many of the examples refer to path integration. When an animal has moved away from its home, or safe place, which is now out of sight, and if, as is commonly the case, it has moved to its present location by an indirect route, its ability to return home, without retracing its movements, involves memory and computation. Etienne and Jeffery (2004) wrote that an organism must continuously gather information on how far, and in which directions it has travelled, and the information on both translations and rotations must be constantly recorded and combined. Alternatively, the animal may navigate based upon remembered landmarks. Either of these methods, or any combination of them, would require a WM. Path integration is a valid indicator of WM that is fairly easily observable, and therefore commonly reported.

An alternative way home that is computationally less demanding for animals, and less adaptive, would be for an animal to retrace its outward route, which would also require a WM. Any animal that returns home, by whatever route, after moving to somewhere from which it cannot sense its home directly, possesses a WM.

The widespread occurrence of WM in chelicerates and mandibulates, which diverged about 550mya (Giribet and Edgecombe, 2019) suggests that their LCA probably possessed WM. It provides some support for the view that WM may have evolved before the separation of arthropods and vertebrates (>600mya).

## 6. WM and attention in octopuses

*Octopus vulgaris*, in their natural environment, make long hunting trips, mainly at night, then return to their home (Kayes, 1973). They use visual spatial information for navigation within their home ranges, and to guide their return from hunting trips, but they do not return by their outward routes. Octopuses that have been displaced from their hunting path return home easily, often following features of the rocky landscape. *O. vulgaris* does not repeat hunts in the same areas of their home ranges on consecutive trips (Mather, 1991). Easily finding their way home, and not repeating the same foraging route on consecutive trips, are evidence that *O. vulgaris* retain a WM of where they are in relation to their home.

Two experimental arrangements involved learned responses of *O. vulgaris* to two different but similar stimuli—the alternative responses were to go left or to go right at the end of a passageway for a reward (Schiller, 1949; Wells, 1964, 1967). Octopuses were able to make correct turns on most occasions even when their response was prevented for up to 2 min after removal of the stimulus, thereby establishing that *O. vulgaris* retained an action-plan in WM.

The animals traversed the passageway with one of their eyes to one wall (Wells, 1964, 1967), or their body oriented to one wall and crawling close to it (Schiller, 1949). In all the experiments, the chosen wall corresponded to the correct choice of direction that would take the octopus to the reward. When the end of this passageway was temporarily closed (Wells, 1964, 1967), only those animals that waited at the closure with their leading eye toward the direction of the reward consistently completed the task successfully. Octopuses that did not wait at the closure but explored the passageway, made random choices which way to go. And, octopuses whose posture was deliberately altered before they reached the end of the passageway, had only a random chance of turning the correct way (Schiller, 1949).

This suggested that the octopus action plan—to turn left, or to turn right—was retained in WM, and maintained by keeping attention to the correct direction by orienting their leading eye or their body towards it. Movement of their attention away from the wall resulted in loss of the action plan from WM.

In another experiment, Octopuses were able to remember where they had seen a crab, though it had been obscured for 30s (Dilly, 1963).

There have also been reports of problem-solving by *O. vulgaris*. Octopuses were able to remove a plastic plug from a glass jar and extract a crab (prey) from inside it (Fiorito et al., 1990). *O. vulgaris* also was able to open an L-shaped container, and to manoeuvre it through a tight fitting hole in a clear plastic partition to get to a piece of shrimp (Richter et al., 2016). Problem-solving, such as in these examples, requires a WM.

In conclusion, the evidence indicates that *O. vulgaris* possesses WM, which can contain information or an action plan. Attention to the next stage of an action plan is necessary for octopuses to retain their action plan in WM.

Some other molluscs have been reported as exhibiting behaviour that requires the presence of WM. Example behaviours are: remembering prey that were out of sight; inhibition of return; direct return to their home; conditional discrimination; delayed gratification; and predicting times and locations that prey would be available:

- Octopuses: *O. bimaculatus* (Ambrose, 1982), *O. bimaculoides* (Boal et al., 2000; Hvorecny et al., 2007), *O. cyanea* (Forsythe and Hanlon, 1997)
- Cuttlefish: *Sepia officinalis* (Sanders and Young, 1940; Karson et al., 2003; Alves et al., 2007; Jozet-Alves et al., 2013; Billard et al., 2020; Schnell et al., 2021)
- Nautilus: *N. pompilius* (Crook et al., 2009).

These reports provide evidence that WM is possessed by some coleoids and nautilus, but there are many other molluscs for which no data are available. However, according to Shigeno et al. (2018), in early development the nervous system of all molluscs have three domains that are comparable to the mammalian forebrain, midbrain, and spinal cord. This suggests the ancestors of all molluscs possessed mental functions that required these complex neural systems, for which they would require a WM. However, direct evidence for inheritance of WM by molluscs since their LCA is limited at the present time.

## 7. WM in *Caenorhabditis elegans*?

It is likely that the nematode, *C. elegans*, possesses WM. *C. elegans* were placed at the base of the stem of a T-maze, and timed to reach a food reward in one arm of the maze (Qin and Wheeler, 2007). The test was repeated, with the reward in place, and it was found that *C. elegans* reached the reward more quickly on each repeat. The authors considered that these results were evidence of associative learning by *C. elegans*. This would be an operant-conditioning type association, because an “emitted” behaviour was being rewarded (Reber, 1995). However, this seems an unlikely explanation, because it would have required single event conditioning of a complex sequence of actions, and it is more likely that *C. elegans* were actually exhibiting signs of an action plan in WM. Returning to a place where an animal previously found food is common WM-based behaviour.

In a similar experimental setup, *C. elegans* were individually placed at the base of the stem of a T-maze, with the end of one arm containing a food reward (Gourgou et al., 2021). The “worms” moved along the floor or wall of the maze. Those that found the food, were tested again with no reward in the arm of the maze, hence, no chemosensory cues. 71% turned to the previously correct side, and, of those “successful” in an unrewarded trial, 75% made the same floor or wall choice as they had in the rewarded trial. The authors stated that the *C. elegans* were able to retain a memory of the behaviour that led them to the reward location for up to 8 min. The authors considered their findings were evidence of a form of WM.

Gourgou et al. (2021) initially used mazes with a coarse surface texture. When they repeated the experiment using T-mazes with very smooth surfaces, *C. elegans* were unable to preferentially choose the correct side of the T on the unrewarded second run. This suggested that *C. elegans* had been using tactile feedback from the rough surface to retain attention on the task in the first series of tests, as would be expected if they were using an action plan in WM.

There is evidence that *C. elegans* has STM and LTM (Ackley, 2019), which can be used to determine responses (requiring WM), and that it shares some memory mechanisms with vertebrates (Rose and Rankin, 2001; Rankin, 2004). Rankin (2004, p. R618) wrote that:

*C. elegans* is capable of integrating and remembering experiences across different sensory modalities [sight, vision, smell, touch, temperature, etc.]. *C. elegans* has a remarkable ability to learn about its environment and to alter its behavior as a result of its experience.

It is likely that the conclusion of Gourgou et al. (2021) was correct, and that *C. elegans* does possess a WM. I have included *C. elegans* in Table 3, which is a summary of data for humans, archerfish, salticids, honey bees, octopus, and *C. elegans*.

## 8. WM and attention in two deuterostomes and four protostomes

Humans and archerfish are deuterostomes, but salticids, bees, octopus and nematodes are protostomes. The defining difference between protostomes and deuterostomes is the order of development of the gut openings: in protostomes the mouth forms first; in deuterostomes the mouth opening forms second. Protostomes and deuterostomes also differ in the location of the principal nerve cord: protostomes have a ventral nerve cord, deuterostomes have a dorsal nerve cord (De Robertis, 2008).

The main conclusions concerning WM and attention in the two types of deuterostomes and four types of protostomes are summarised in Table 3. (I use “types” because the archerfish data and salticid data each refer to more than one species.) The information in Table 3 shows the close similarity between the properties of WM and attention in the six animal types. This similarity suggests the possibility of common evolutionary origin.

### 8.1. The phylogenetic relationships between animals with reported WM

**Figure 2:**
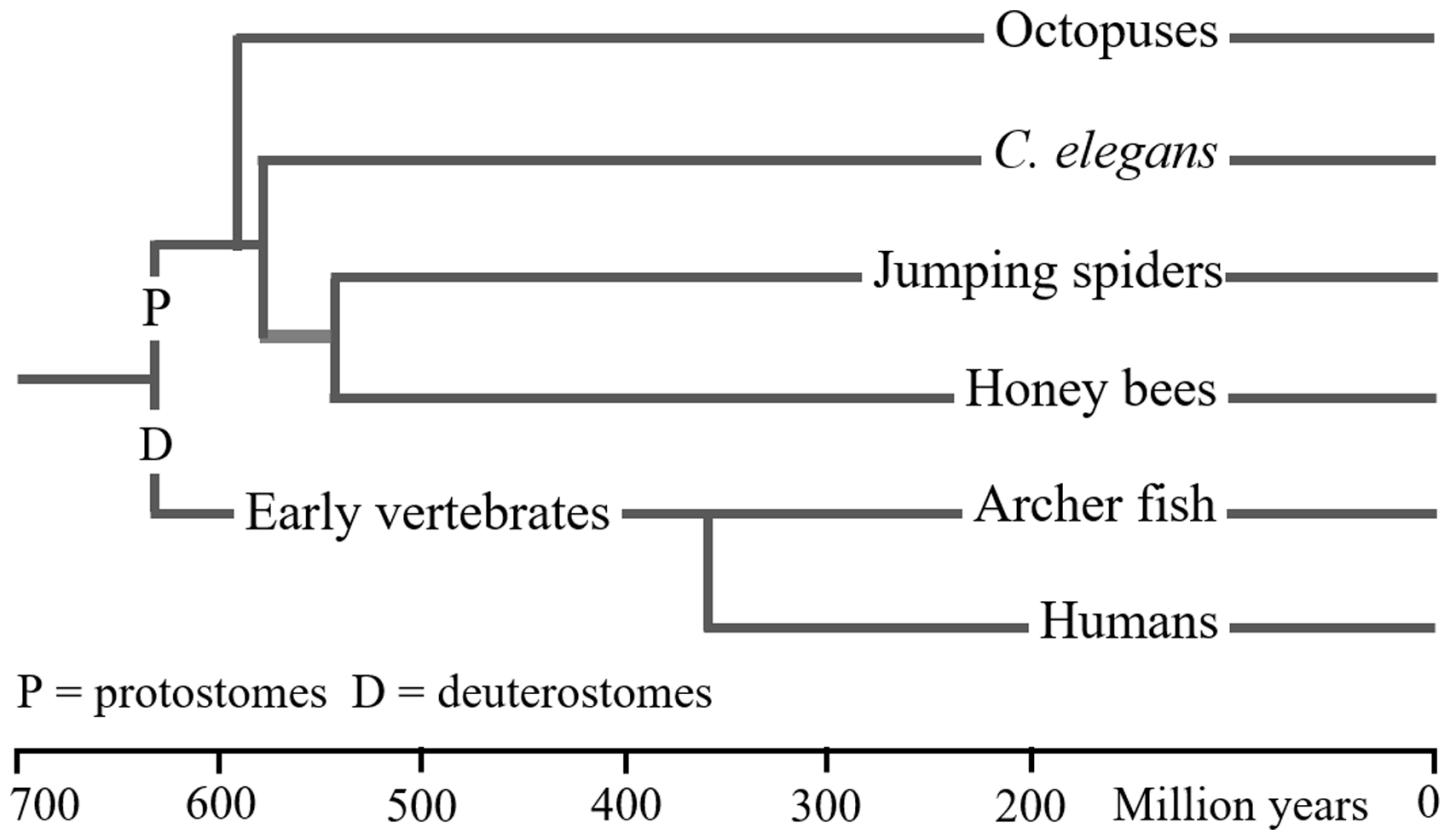
Phylogenetic relationship of six animal. This figure provides only a general impression of the dates at which these animals first evolved. As noted in Figure 1, there are various factors that result in large differences between the dates presented in different reports (Cunningham et al., 2016). I have used dates from a single recent publication (dos Reis et al., 2015). LCA dates are shown as vertical lines. The order in which molluscs and nematodes from the precursors of arthropods is supported (but not dated) by Moroz et al. (2006).

Table 3 summarises the properties of WM in the six animal types, and Figure 2 gives an approximate measure of the time since the common ancestor of these species was alive. The LCA of humans (or archerfish) and salticids (or honey bees) are claimed to have lived about 640mya. Yet, the properties of WM in these species is almost identical despite almost 1.3 billion years of evolutionary separation. Either WM has existed since the LCA, and any changes that occurred were common to the different species, or WM evolved multiple times in very similar forms.

The evidence for WM having been inherited, since the LCA of the species in Table 3, by molluscs and nematodes is very limited. But, as shown in Figure 2, it is reported that vertebrates diverged from protostomes before molluscs and nematodes diverged from other protostomes. Therefore, if vertebrates (and arthropods) inherited WM and attention from the LCA, molluscs and nematodes would have too, provided the phylogenetic dating is in the correct time order.

## 9. Aspects of brain structure conserved since the LCA of protostomes and deuterostomes

There are currently two views on the evolution of brains (Northcutt, 2012; Liebeskind et al., 2016). The dominant view is that the LCA of protostomes and deuterostomes possessed a simpler form of the tripartite brain—consisting of forebrain, midbrain and hindbrain—that is possessed by vertebrates and arthropods (see references below), but that in some later species the brain has degenerated. The alternative view is that the LCA possessed only a neural net, and that the tripartite structure has separately evolved on multiple occasions (Martín-Durán et al., 2018). The factors which allow this difference of view to persist include: 1. no fossil has yet been found that can be attributed to the LCA, which might resolve this issue; 2. some genes are utilised in different developmental processes and may have been present in the LCA without it having a brain; 3. neural structures can be lost over time, just as other evolved properties can be lost.

The hypothesis presented here, that WM has been inherited since the LCA of protostomes and deuterostomes, depends partly upon the correctness of the view that the LCA possessed a tripartite brain, or at least a somewhat complex neural system. This is because a brain or complex neural system could only have evolved because it was capable of mental processing that was more flexible and adaptive than S-R, and this would require a WM. By contrast, a nerve net would only possess a WM if it were able to store information of some kind and was capable of manipulating this information to determine behaviour beyond the inhibition and sensitisation of S-R actions.

There are many reports, based upon various lines of evidence, that genes which control the development of brain structure in protostomes and deuterostomes (primarily in insects and mammals) have been conserved since the LCA (Arendt and Nübler-Jung, 1999; Hirth and Reichert, 1999; Ghysen, 2003; Hirth et al., 2003; Lichtneckert and Reichert, 2005; Arendt et al., 2008; Nomaksteinsky et al., 2009; Hirth, 2010; Holland et al., 2013; Fiore et al., 2015; Wolff and Strausfeld, 2016; Bridi et al., 2020).

The tripartite brain—comprising the forebrain, midbrain and hindbrain—exists in many protostomes and deuterostomes. These three parts of the brain in the two clades are different in gross structure (though sometimes less so during early development) but they are similar in function (Hirth and Reichert, 1999; Lichtneckert and Reichert, 2005; Hirth, 2010; Tomer et al., 2010; Fiore et al., 2015; Shigeno et al., 2018; Bridi et al., 2020).

It has been reported that genes can be transferred between an insect (a protostome) and mammals (deuterostomes) and cause relatively normal brains to develop where a homologous gene, that is essential to proper brain development, is absent from the recipient. *Drosophila* genes whose homologues were missing from mutant mice were found to effect a near complete rescue of the mouse brain (Hanks et al., 1998). Brain defects in mutant *Drosophila* were rescued using homologous human genes (Nagao et al., 1998). Brain structures in mutant *Drosophila* were rescued by either *Drosophila* or human gene homologs (Leuzinger et al., 1998). These results indicate that the genetic mechanisms underlying brain development in insects and mammals are evolutionarily conserved (Hirth and Reichert, 1999).

It is surprising that these genes have changed so little over the enormous evolutionary time that separates mammals and insects. This has been explained on the basis that once a certain level of neural complexity, of structures and functions, has evolved, any genetic change is likely to be deleterious (Ghysen, 2003). If that is correct, genetic changes would be selected against unless they have essentially the same properties as the genes they replace.

Genetic analyses in vertebrates (Boyl et al., 2001), arthropods (Hirth et al., 2003), cephalopods (Shigeno et al., 2018), urochordates (Wada et al., 1998), nematodes (Aspöck et al., 2003), and annelids (Denes et al., 2007; Tomer et al., 2010), support the view that genes which control development of the CNS have been conserved since the LCA of protostomes and deuterostomes. This may indicate that the LCA possessed a brain of some kind, and that its structure was similar to those of some modern protostomes and deuterostomes.

In a review of the evidence for single or multiple evolution of brains, Holland et al. (2013, p. 1) wrote that “The bulk of the evidence indicates that a CNS evolved just once—in the ‘ancestral bilaterian’, which was prior to the divergence of protostomes and deuterostomes.” It is likely that the LCA of protostomes and deuterostomes already possessed a tripartite brain of modest complexity (Roth, 2015).

Genetic analyses of relevant genes in cephalopods (Shigeno et al., 2018) support the view that they were inherited since the common ancestor of protostomes and deuterostomes. According to Shigeno et al. (2018) the nervous systems of all molluscs, in early development, have three domains that are comparable to the mammalian forebrain, midbrain and spinal cord. From Figure 2 it can be seen that molluscs are believed to have diverged from other protostomes after the divergence of vertebrates. Therefore, if vertebrates inherited the complex of brain development genes, molluscs must also have inherited them. Subsequently, the brains of some molluscs may have degenerated because they were no longer needed for the lifestyles that the species had adopted.

Evidence of WM has been reported for only one nematode, *C. elegans*, which might suggest independent evolution of WM in its forebears. But evidence that nematodes inherited genes associated with brain development from the LCA (Aspöck et al., 2003), and that nematodes diverged from other protostomes later than vertebrates (Figure 2) suggests that early nematodes could also have possessed a tripartite brain.

Evidence that genetic structures controlling the development of neural structures existed prior to the LCA is important because it implies some level of cognitive activity existed at that time. Based on commonalities between neural structures in insects and mammals, Wolff and Strausfeld (2016) suggested that elements of learning and memory circuits were possessed by the LCA of protostomes and deuterostomes. They also stated that the correspondence of forebrain centres in protostomes and deuterostomes indicates that their LCA possessed an executive brain. Bridi et al. (2020) wrote that conserved mechanisms control brain development and generate circuits for adaptive behaviour in all animals that possess a brain. This would mean that WM, and attention, existed prior to the LCA, because cognitive activity beyond what is needed for S-R actions requires a WM.

## 10. Discussion

*WM* is the retention of information in an “active” state, a condition in which it is available for use by mental processes. *Attention* is a condition in which focal information is detailed (higher density) and non-focal information is low density. (“Focal” information is information that is currently of interest; it is not necessarily visual information.) Attention is a specific kind of localised variation in information density. The difference between focal and non-focal information density increases with the difficulty or importance of the task being processed, which is referred to as the “attentional load”.

Attention is adaptive because enhancement of focal information allows easier identification of objects of interest, and optimum tracking of their movements. Attention is also important because it is the means whereby retained information is held in an active state—attention determines which information is in WM. Attention can activate incoming information or information in STM or LTM. WM and attention are located in relevant storage locations in the brain for sensory or other information.

WM, with attention, is very adaptive for mobile animals because it permits the retention, in an active state, of information that is needed for response selection. This may be to prepare for a predator, or any other threat or opportunity, that is no longer sensed, but may still be nearby; to avoid foraging in an area recently visited, or returning to an area where a danger was recently detected; to return to an area where possible opportunity, such as a mate, or prey, was observed; for retention of steps in an action plan during preparation or activation of the plan; and for all other mental processes that require retention of information, such as problem-solving.

It is important to note that neither WM nor attention are necessarily conscious. Therefore, the possession of these functions by organisms does not indicate that they possess consciousness of any kind. Unconscious attention may activate unconscious WM to resolve matters of concern outside of awareness, including in humans.

The present hypothesis is that WM, with attention, has been inherited by humans, archerfish, and other vertebrates; jumping spiders, honey bees, and other arthropods; and possibly octopus and the nematode, *C. elegans*, since their last common ancestor (LCA). Humans and archerfish are deuterostomes, and jumping spiders, honey bees, octopus, and nematodes are protostomes. The LCA (the most recent shared ancestral species or gene pool) of protostomes and deuterostomes is believed to have lived more than 600mya (Peterson et al., 2008; Edgecombe et al., 2011; Erwin et al., 2011; Northcutt, 2012; dos Reis et al., 2015; Erwin, 2015; Peterson and Eernisse, 2016).

Published reports concerning humans, archerfish, salticids, bees, *O. vulgaris*, and *C. elegans*, indicate that all six possess WM and attention (with some uncertainty about *C. elegans*). Within the limitations of the published data, it appears that the properties of attention-WM possessed by humans are very similar to those of the other species. These species are phylogenetically very diverse within the protostome and deuterostome clades and the “total evolutionary distance” between them is equivalent to billions of years of evolution. Yet the properties of attention-WM appear to be similar across these species.

Other mammals and fish in addition to humans and archerfish, and members of other vertebrate classes; amphibians, reptiles and birds, possess WM. Other arthropod species, in addition to spiders and bees, also possess WM, and a few other molluscs in addition to octopus are known to possess WM.

The possession of WM and attention by these various animal types indicates either that WM and attention independently evolved with very similar properties on multiple occasions, or that these functions have been conserved since their LCA. Or it is possible that some species inherited from a common ancestor, but WM independently evolved in the ancestors of other species.

There is evidence that genetic structures which control the development of brains have been conserved since the LCA of protostomes and deuterostomes. Essentially the same combination of genes is central to the development of brains in vertebrates, arthropods, cephalopods, urochordates, nematodes, and annelids. There have been changes in these various genes, but it seems that they continue to perform essentially the same functions during brain development, and there is some evidence that they are exchangeable between insects (protostomes) and mammals (deuterostomes), with near normal development of the recipients’ brains. This appears to be clear evidence that genes which control brain development have been conserved.

Genes that control brain development only evolve and are conserved because the brains generate flexible, adaptive behaviour. Brains could not have evolved without the parallel evolution of flexible behaviour. But flexible behaviour requires that information be retained in an appropriate state for use by the processes that generate these behaviours, and this would be as WM. WM is the essential mechanism that underpins all flexible mental processes. It is proposed that the evolution of WM was a very early step in the in the evolution of flexible processing, which preceded the evolution of brains. This proposal gains some support from evidence that the nematode, *C. elegans*, with only 302 neurons (White et al., 1986), very likely possesses WM and attention.

It is likely that WM and attention have been conserved in many species since the LCA of protostomes and deuterostomes or earlier. This does not imply that all protostomes and deuterostomes have WM, because evolutionary processes can result in species losing functions that their ancestors possessed where their lifestyle is such that possession of WM is not necessary for continuance of the species. For such species, loss of WM could be adaptive because it would prevent unnecessary expenditure of resources on a function that was not needed.

Additional observations and experiments are needed to extend the knowledge of WM and attention in vertebrates and arthropods, and test the hypothesis that WM and attention were possessed by their LCA. It is predicted that all species which are descendants of the LCA of vertebrates and arthropods will be found to possess WM, excepting where possession of WM is not necessary for continuance of the species.

If the hypothesis that vertebrates and arthropods inherited WM and attention from their LCA is supported, further research could clarify whether other molluscs, other nematodes, and members of other clades possess WM. This may provide an indication of when WM first appeared in the evolution of animals.

In due course, other mental mechanisms may be found that are common to protostomes and deuterostomes, and likely to have been possessed by their LCA. Discovery of other homologous mental mechanisms could enable a better understanding of the nature and lifestyle of the species that was the LCA of protostomes and deuterostomes—our ancestors from long ago.

## Data availability statement

The original contributions presented in the study are included in the article/supplementary material, further inquiries can be directed to the corresponding author.

## Author contributions

The author confirms being the sole contributor of this work and has approved it for publication.

## Funding

All costs associated with this research were paid by the author. No institutional, or other source of funding, was involved.

## Conflict of interest

The author declares that the research was conducted in the absence of any commercial or financial relationships that could be construed as a potential conflict of interest.

## Publisher’s note

All claims expressed in this article are solely those of the authors and do not necessarily represent those of their affiliated organizations, or those of the publisher, the editors and the reviewers. Any product that may be evaluated in this article, or claim that may be made by its manufacturer, is not guaranteed or endorsed by the publisher.

## Footnotes

^1^Mind has been defined as the mechanisms in the brain which select, initiate, and control behaviour (Chalmers, 1996; Earl, 2019). However, this definition excludes animals that do not possess a localised concentration of neural structures which might reasonably considered to be a brain. It is proposed that mind be redefined as neural mechanisms that determine behaviour. This definition would include response systems in animals with neural networks rather than a brain, but would also extend the meaning of mind to include S-R actions, in agreement with Roth (2013).
